# Phoneme Representation and Articulatory Impairment: Insights from Adults with Comorbid Motor Coordination Disorder and Dyslexia

**DOI:** 10.3390/brainsci13020210

**Published:** 2023-01-27

**Authors:** Rebecca Marchetti, Serge Pinto, Laure Spieser, Marianne Vaugoyeau, Eddy Cavalli, Abdessadek El Ahmadi, Christine Assaiante, Pascale Colé

**Affiliations:** 1 Laboratoire de Neurosciences Cognitives (LNC), French National Centre for Scientific Research (CNRS), Aix-Marseille University, 13007 Marseille, France; 2 Laboratoire de Psychologie Cognitive (LPC), French National Centre for Scientific Research (CNRS), Aix-Marseille University, 13003 Marseille, France; 3 Federation de Recherche 3C, French National Centre for Scientific Research (CNRS), Aix-Marseille University, 13003 Marseille, France; 4 Laboratoire Parole et Langage (LPL), French National Centre for Scientific Research (CNRS), Aix-Marseille University, 13100 Aix-en-Provence, France; 5 Laboratoire d’Etude des Mécanismes Cognitifs (EA3082), University Lumière Lyon 2, 69007 Lyon, France

**Keywords:** dyslexia, adulthood, comorbidity, articulation, phonemic representation quality

## Abstract

Phonemic processing skills are impaired both in children and adults with dyslexia. Since phoneme representation development is based on articulatory gestures, it is likely that these gestures influence oral reading-related skills as assessed through phonemic awareness tasks. In our study, fifty-two young dyslexic adults, with and without motor impairment, and fifty-nine skilled readers performed reading, phonemic awareness, and articulatory tasks. The two dyslexic groups exhibited slower articulatory rates than skilled readers and the comorbid dyslexic group presenting with an additional difficulty in respiratory control (reduced speech proportion and increased pause duration). Two versions of the phoneme awareness task (PAT) with pseudoword strings were administered: a classical version under time pressure and a delayed version in which access to phonemic representations and articulatory programs was facilitated. The two groups with dyslexia were outperformed by the control group in both versions. Although the two groups with dyslexia performed equally well on the classical PAT, the comorbid group performed significantly less efficiently on the delayed PAT, suggesting an additional contribution of articulatory impairment in the task for this group. Overall, our results suggest that impaired phoneme representations in dyslexia may be explained, at least partially, by articulatory deficits affecting access to them.

## 1. Introduction

Developmental dyslexia (hereafter, dyslexia) is a neurodevelopmental disorder affecting approximately 5–10% of the population [1,2], depending on the transparency of the orthographic system [3]. Dyslexia results in significant and persistent decoding and word-reading difficulties, which may also impair reading comprehension and lead to poor spelling performance [4]. Over the last 20 years, numerous studies have attempted to identify the causes of dyslexia and, following the work by Pennington [5], have led to a multifactorial conception of these causes [6,7,8,9,10,11,12] which provides an explanatory framework for the frequently reported comorbidities between dyslexia and other neurodevelopmental disorders [13]. Moreover, many studies have shown that the symptoms of dyslexia persist into adulthood (e.g., [14,15]). In this context, the main goals of the present study, which examined a sample of adults with dyslexia for whom a persistent and stabilized phonological impairment had been consistently reported (for English: [16]; for French: [15,17]), were (1) to investigate whether motor coordination disorders (hereafter, motor disorder) may also involve the oro-motor sphere, suggesting co-occurring articulatory deficits in adults with dyslexia, and (2) to study the relationship between articulatory impairment and phonemic awareness deficit in dyslexia, given that phonemic awareness is a crucial component involved in skilled reading. More generally, this study also aimed to provide evidence about the nature of the phoneme deficit in dyslexia.

### 1.1. Single vs. Multiple-Deficit Hypotheses in Dyslexia

Single deficit hypotheses such as the auditory hypothesis [18,19], the visual hypothesis [20,21], or the cerebellar/motor theories [22,23,24,25] have been proposed as possible causal explanations of dyslexia. For a number of decades, the phonological hypothesis has been considered to be an important explanation of the origin of dyslexia within a unitary, single-factor approach (see [13] for a review). Impaired performance in tasks involving different kinds of phonological processing has been consistently reported in individuals with dyslexia, that is, in phonemic awareness (in children: [26]; [27]; in adults: [15]), in short-term verbal memory (in children: [28]; in adults: [29]), in rapid naming (the deficit remains stable from childhood to adulthood, see [30] for a meta-analysis), and in pseudoword decoding tasks (in children: [31]; in adults: [15]). The fact that this type of phonological deficit is systematically reported in adults with dyslexia suggests that the phonological disorder stabilizes in adulthood [14,32,33,34], although, the specific nature of the phonological deficit is still a matter of debate [35]. Some researchers consider that imprecise phonemic representations are the cause of phonological disorders in dyslexia [36,37,38], while others suggest that these representations remain intact but that access to them (or activation of them) is delayed [39,40,41,42].

The shift from a single- to a multiple-deficit conception of dyslexia has found some support in reports of frequent symptom comorbidity in dyslexia. Rates of comorbidity vary widely, but it is expected that about 40% of children with a reading disorder will also present with another neurodevelopmental disorder [43]. For example, between 25 and 40% of individuals with attention deficit and hyperactivity deficit (ADHD) have dyslexic problems and vice versa [44]. Regarding the comorbidity between dyslexia and developmental motor coordination disorder (hereafter, DCD), this ranges from 30 to 50% [45,46]. In this article, the term “comorbidity” refers to “the co-occurrence between two (or more) disorders in the same individual” in line with Snowling, Hulme, and Nation (2020) [43], page 505. DCD is a neurodevelopmental disorder that affects the acquisition and execution of coordinated motor skills and cannot be explained by mental retardation or neurological impairment [4]. Although research has reported that dyslexia-DCD comorbidity occurs in about 1 out of 2 dyslexic children [27], its prevalence seems to be reduced in adults (1 out of 4; see, for example, [34]). Recently, we administered the French adaptation [47] of the MABC-2 extended version (a reference test used to evaluate potential DCD [48]) and reported that 27% of university students from a sample with a prior diagnosis of dyslexia were affected by motor impairment, while only 5% of the control reader group were affected [49]. As we have already pointed out [49], sensorimotor deficits in dyslexia can include postural, oculomotor, motor coordination, and implicit motor learning disorders in dyslexic children and adults [27,34,50,51]. More recent research on dyslexia ([52], for a recent review), and on motor comorbidities, have, for example, addressed dysfunctions in writing abilities [53,54] and even rhythmic motor competencies [55]. Nevertheless, there is still a consensus that motor difficulties are present in part of the dyslexia population, albeit to a larger extent in children than adults [27,34].

### 1.2. The Relationship between Phonemic and Motor Deficits in Adults with Dyslexia: The Link between Speech and Articulatory Skills

Earlier developmental studies have reported that articulatory skills are closely linked to both the phonological system and the lexicon, as they overlap and develop simultaneously at the onset of language development [56,57] and in the following years (for a recent account, see [58]). Thus, according to Liberman [59], articulatory gestures would provide the basic architecture for young children’s speech perception, representation, and production. In other words, articulatory gestures—*the motor patterns of the speech production organs corresponding to individual phonemes*—may constitute the information underpinning speech sounds, thereby structuring the speech system of adults [60]. Because phoneme representations are also based on articulatory gestures, they are therefore likely to influence reading-related competencies such as phonemic awareness skills (see [61]). Liberman’s hypothesis fits well with motor theories of speech perception [62], which propose that articulatory gestures are not only the elemental events of speech production but are also critical for speech perception, with the two components (perception and production) of the speech system being parts of the same process. The perception of speech sounds would thus be coupled with the concomitant set of articulatory gestures (mouth, lip, velum, and larynx), as pointed out by a number of physiological and neuro-imaging studies on the implications of the motor and articulatory system for language perception [63,64,65,66,67,68]. More precisely, the findings obtained in physiological and neuroimaging experiments [69,70,71] for reviews) and the propositions of motor theories of speech such as that developed, for example, by Skipper and Colleagues [56], can help to provide a precise interpretation of the processing of articulatory features during the perception of speech sounds, as can the models of the motor control of speech [72,73,74]. At a very schematic level, Skipper’s model holds that auditory information is first processed by the auditory cortex, which generates phonemic hypotheses that are then projected to the left inferior frontal gyrus and matched to the articulatory goals that may most typically be the origin of these hypothetical phonemes. The ventral central premotor cortex, acting via the primary motor cortices, then simulates the underlying motor commands, on the basis of which it produces efference copies (i.e., internal copies of the motor commands that make it possible to simulate and anticipate the sensory consequences of these commands). These copies are then transmitted to the auditory cortex to constrain the phonetic interpretation of the phonemic hypothesis. Further support for this theory has been provided by Studdert-Kennedy [75] within a conception that incorporates the potential role of mirror neurons in speech perception [76].

Past research has provided extensive data showing that phonemic skills are a critical determinant in learning to read [13,77], with children suffering from dyslexia typically experiencing phonological processing deficits [78] that persist into adulthood [14,34,79]. However, as mentioned above, dyslexia is frequently associated with comorbid motor deficits. Three seminal studies attempted to explain how phonological and motor disorders cause reading impairments in dyslexia. These studies used a wide range of tasks that primarily assessed postural control and manual dexterity in adults and children [27,34,80]. They reported that, whereas phonological skills significantly predicted literacy skills, this was not the case for motor skills. Importantly, White and collaborators [80] also reported that motor skills did not predict any variance in phonological skills. One of the main problems with these studies is that they focused on general axial motor skills (postural stability, stork balance, heel-to-toe walk) that may be quite different from those involved in oral language skills and in learning to read [80]. Thus, some studies have provided data showing that articulatory speech information is crucial to the phoneme representations involved in learning to read and in phoneme awareness tasks [61,81,82]. Indeed, Kent [83] reported that the physical growth of the vocal tract is not complete until adolescence. From birth to adulthood, the production of speech sounds is likely to reflect continuous articulatory and acoustic adjustments that occur as the production system matures [60] and this influences phoneme awareness performances in some way. Thus, it can be inferred that the assessment of articulatory speech skills might be a more relevant way of investigating the involvement of motor skills in phonemic awareness tasks and, indirectly, in reading.

To our knowledge, articulatory speech deficits have only very rarely been investigated in individuals with dyslexia, with no distinction being made between participants with and without motor disorders. A few studies have looked at articulatory deficits in individuals with dyslexia and reported impaired performance compared to control readers. For example, significant problems regarding the speed of articulatory movements involved in speech production have been found in participants aged 6–11 years [84], 9–14 years [85], and 13–16 years [86]. Similar results were reported by Bradshaw, Woodhead, Thompson, and Snowling [87], who used a sentence repetition task from the NEPSY [88] to evaluate deficits in oro-motor skills in adults with dyslexia. Moreover, Griffiths and Frith [89] used an articulatory awareness task in which adults with dyslexia had to repeat orally presented phonemes and match them with schematic drawings of their articulations. However, these two studies with adults did not distinguish between performances in light of the DCD comorbidity of the participants. Moreover, they used different kinds of tasks to assess the articulatory components of speech production. In particular, the sentence repetition task [88] does not exclude the possibility that lexical knowledge may also be activated as a compensatory mechanism (study [90] has the same drawbacks). At the same time, the articulatory awareness task [89] does not make it possible to dissociate the deficits arising from implicit or explicit (conscious) access to articulatory codes.

### 1.3. The Present Study

The first aim of this study was therefore to use a diadochokinesis (hereafter DDK) task to determine whether the fine and gross motor deficit reported by [49] is also associated with an articulatory/orofacial deficit in adults with dyslexia and developmental motor coordination comorbidity. Diadochokinetic performance is measured in terms of the time needed to process motor gestures that are necessary for the production of specific and frequent syllables; as such, it can be considered a model for assessing the spatial programming of speech production (see [84]) and is related to individuals’ articulatory skills. In DDK tasks, participants are asked to repeat meaningless syllables (e.g., a single syllable /puh/, /kuh/, and /tuh/ or a syllable sequence /puh-kuh-tuh/ or /pa-ta-ka/), produced by combining vowels and consonants, for a period of time and under time pressure. According to studies conducted with adults [87,88,89], articulatory deficits in a DDK task are expected in both adult groups with dyslexia, with the impairment being exacerbated in the comorbid group.

Given that articulatory skills contribute to the quality of phonological/phonemic representations [91], the second aim of this study was to identify the potential effects of this articulatory deficit on oral reading-related skills, such as phonemic awareness skills. To this end, we administered a classical phonemic awareness task (hereafter, classical PAT) and a modified version of this task in order to highlight articulatory speech problems. The classical version took the form of an initial phoneme deletion task with pseudowords, a task widely used to assess phonemic skills related to reading achievement [13]. Downing and Caravolas [92] used a combination of measures, such as phoneme deletion, phoneme blending, and rapid automatized naming, to test the impact of dyslexia–DCD comorbidity on phonological processing. They found that children with literacy impairment and comorbid literacy/motor disorders underperformed compared to children without literacy disorders, with the two dyslexic groups achieving similar performances. In our classical PAT task, we expected to observe results similar to those reported by Downing and Caravolas [92], meaning that the time pressure of the task should not produce any difference between the two groups with dyslexia. To investigate potential articulatory disorders in comorbid participants in more detail, the task was also administered in a modified form referred to as the delayed phonemic awareness task (hereafter delayed PAT). In this task, there was a longer interval between the end of the pseudoword presentation and the participant’s answer (this interval was pre-determined for each participant individually). This was intended to facilitate both access to phonemic representations of pseudoword strings and the programming of the articulatory speech codes needed in order to produce the answer. We, therefore, expected all three groups to become more efficient, although the potentially exacerbated oro-motor deficits of the comorbid group were expected to result in a smaller facilitation effect in this group than in the non-comorbid dyslexic group. More generally, we expected the comparison of performances on the two versions of the PAT to provide some information on the impact of articulatory disorders on the quality of phoneme representations in dyslexia and thus to help resolve the debate about the nature of the phoneme deficit in dyslexia (imprecise phoneme representations and/or delayed access to these representations). If we assume that the phonemic representations of dyslexic participants are imprecise/degraded then we would expect to observe significantly poorer performance in both groups of dyslexics than in the control group on the two versions of the PAT and in particular in the delayed version, in which the speed of access to phonemic representations (or their speed of activation) is controlled.

The third aim was to explore the relationship between phonemic and articulatory skills in greater depth. We hypothesized that articulatory gestures may constitute the information underpinning and structuring of the adult speech system. Given the persistent phonemic processing difficulties of adults with dyslexia and the role of articulation in the development of phonemic representations, articulatory performance should contribute more to explaining the classical PAT scores in the dyslexic sample.

## 2. Method

### 2.1. Participants

The sample set for this study consisted of 111 university students, aged 18–29, This sample (see [49]) contained 59 skilled readers with no history of reading disabilities (hereafter SR: 18 men, 41 women; mean age: 21.7 years ± 1.9 years) and 52 participants with developmental dyslexia (hereafter DYS: 19 men, 33 women; mean age: 21.7 years ± 2.3 years). The DYS group was divided into two subgroups, one with motor impairment (hereafter DYS-CoM: *n* = 14, 5 men, 9 women) and one without motor impairment (hereafter DYS-noCoM: *n* = 38, 14 men, 24 women). SR were recruited from the university population through advertisements and information sessions at Aix-Marseille University (AMU), and DYS were recruited through the Mission Handicap of AMU (a disability support service, part of the university medical service). The experiment was conducted in accordance with the Declaration of Helsinki and with the understanding and written consent of all participants. The experiment was approved by the local ethics committee of AMU.

Motor impairment was assessed using the extended version of the M-ABC 2 [48], a reference test [46,50,93] which is widely administered to young adult populations [94,95,96,97] (see [49], for more information). The M-ABC 2 consists of eight tasks, grouped into three categories: manual dexterity, aiming and catching, and balance. A score below or equal to the 5th percentile is considered to indicate a sensorimotor deficit. Assuming that the ceiling levels could be reached more easily due to the more advanced maturation of the motor system in our participants (young adults), the <5th percentile can be considered as a conservative cut-off for motor impairments. Participants with a score below the 5th percentile were considered to present with a motor impairment. As can be seen in Table 1, both DYS groups and SR were matched on age (F < 1), years of higher education (F < 1), vocabulary (F < 1) (as measured by the EVIP scale [98], the French adaptation of the Peabody Picture Vocabulary Test-Revised, PPVT-R), and non-verbal IQ (Raven’s Matrices [99]) (F(2, 108) = 1.82; *p* < 0.17). They were also matched on sex (SR: 69% women, DYS-noCoM: 63% women, DYS-CoM: 64% women). DYS and SR groups differed significantly on reading scores assessed using a French *reading test* standardized for adults (*l’Alouette*, [15]). The Alouette test [100] consists of 265 words formed into grammatically and syntactically correct but meaningless sentences, thus making it impossible to refer to any background context. The participants were asked to read the text aloud as rapidly and accurately as possible for a maximum of three minutes. A reading efficiency score was calculated based on reading time (time taken to read the text) and accuracy (number of words correctly read) (see [15] for more details). The ANOVA conducted on the Alouette data (reading fluency) yielded a significant effect of group (F(2, 108) = 71. 96, *p* < 0.001, η^2^ = 0.58). The contrast analyses indicated that DYS-noCoM performance did not differ from DYS-CoM (*t*(108) = 0.425, *p* = 0.672) and that the two DYS groups differed significantly from the SR group (*t*(108) = 11.36, *p* < 0.001).

The participants with dyslexia had been diagnosed *in a Center for the Diagnosis of Learning Disabilities* (Centre de Référence des Troubles des Apprentissages) during their childhood or adolescence, and 76% of them had received learning support for an average of 4.27 years (SD = 3.74). They all reported major difficulties in learning to read during childhood and/or adolescence. These difficulties were confirmed in adulthood using the French translation of the Adult Reading History Questionnaire (ARHQ) scores. The ARHQ was developed by Lefly and Pennington [101] and takes the form of a self-reported questionnaire consisting of 23 Likert-scale items, including questions on earlier difficulties in the acquisition of reading skills as well as on current reading behaviors. The ANOVA conducted on the ARHQ scores yielded a significant effect of group (F(2, 108) = 130.337, *p* < 0.001, η^2^ = 0.71). The contrast analyses indicated that DYS-noCoM performance did not differ from DYS-CoM (*t*(108) = 0.375, *p* = 0.71), and that the two DYS groups differed significantly from the SR group (*t*(108) = 15.225, *p* < 0.001).

All participants were native French speakers with normal or corrected-to-normal vision. They had no auditory or neurological/psychiatric disorders and had a non-verbal IQ within the normal range (above the 75th percentile). Because the current study relied heavily on auditory tasks, the auditory skills of each participant were assessed by identifying their hearing thresholds by means of a screening audiometer (Resonance R17A, MRS, Italy). Pure tone averages were detected at three frequencies: 0.5, 1, and 2 kHz, for both ears. The ANOVA yielded no significant effect of either group or laterality (left ear vs. right ear) with F < 1, but did reveal a significant effect of frequencies (F(2, 216) = 192.15, *p* < 0.0001). However, neither the group × frequency interaction (F < 1) nor the group × laterality interaction (F(2, 108) = 1.6103); *p* = 0.206) reached significance.

**Table 1:** Means (and standard deviations) for chronological age, educational level, vocabulary (raw scores), Raven’s matrices (raw scores), reading score, and Adult Reading History Questionnaire (ARHQ) scores for participants with dyslexia and without motor impairment, (DYS-noCoM) and with motor impairment (DYS-CoM) as well as for control skilled readers (SR).

	SR		DYS-noCoM		DYS-CoM	
	M	SD	M	SD	M	SD
Chronological age (years)	21.7	1.9	21.7	2.3	21.6	1.5
Years in higher education	3.6	1.6	3.5	2.1	3.2	1.3
Non-verbal IQ (RAVEN’s matrices)	49.6	4.8	48.2	5.2	47	5.3
Vocabulary skills (EVIP)	36.9	5.1	36.3	4.7	36.6	5.4
Reading fluency (efficiency) (Alouette)	559.7	91	368.1	75.4	356.7	85.4
ARHQ/0.92	0.30	0.09	0.59	0.09	0.60	0.10

Note: With regard to the ARHQ score, according to [102], a score above the cut-off score of 0.43 indicates significant reading difficulties.

### 2.2. Experimental Procedure

#### 2.2.1. Reading Tasks

A *one-minute word reading test* was performed to assess the efficiency of the orthographic reading procedure. It consisted of 120 bisyllabic words presented on a printed sheet containing six words per line. The words were between four and nine letters in length (mean = 6.4; sd = 1.29), were of low to high frequency (mean = 28.6; sd = 43.4) and were selected using the *lexique.org* database [103]. Participants were instructed to read the written words aloud as fast and accurately as possible within a 1 min time limit. An efficiency score that took account of both accuracy (A) and reading time (RdT): (A × 60)/RdT was then calculated for each participant individually.

*A two-minute pseudoword reading test* was also administered to assess the efficiency of the participants’ decoding skills (i.e., phonological procedure). It consisted of 116 pseudowords that varied in the number of syllables (60 monosyllabic, 60 disyllabic) and length (mean = 5.5; sd = 0.5). These were presented on a printed sheet containing six pseudowords per line. Participants were instructed to read the written pseudowords aloud as fast and accurately as possible within a 2 min time limit. Efficiency scores were calculated for each participant: (A × 120)/RdT.

In the *connected text reading fluency test*, participants were instructed to read aloud as fast and accurately as possible within a 1 min time limit, while also respecting the punctuation marks. The text was taken from *“The red silk scarf”* (L’écharpe de soie rouge [104]), a short narrative literary French text from which we selected the first 337 words (17 sentences). The final efficiency score corresponded to the number of words read correctly in 1 min.

#### 2.2.2. Articulatory Diadochokinesis Task

It is possible to use oral DDK tasks with sequential or alternating motion rates in order to distinguish between motor and linguistic processes and to avoid potential top-down confounding effects. In these tasks, the participants have to repeat single syllables or meaningless sequences of syllables (e.g., /pataka/) at a normal or accelerated rate either during a single outbreath or for a determined duration (e.g., 30 s) [105,106,107]. In our DDK task, participants were required to produce the tri-syllabic pseudoword /pataka/ as fast and accurately as possible for 30 s at a pitch and volume they felt comfortable with. The syllables chosen for this DDK task made it possible to assess the three major orofacial articulatory organs, that is, the lips (/pa/), the tip of the tongue (/ta/), and the dorsum of the tongue (/ka/). Following the guidelines of [108] and according to the methodology used in [109], it is possible to calculate three types of indicator: 1. The articulation rate (number of syllables/minute), which makes it possible to estimate the quality of supralaryngeal articulation; 2. The speech proportion (i.e., ratio between the cumulated speech durations and the total session time); and 3. The pause proportion (i.e., ratio between the cumulated pause durations and the total session time), which provides information on the respiratory control required for speech production. The task was recorded and audio files of the participant’s productions were pre-processed and analyzed using dedicated software (Praat http://www.fon.hum.uva.nl/praat/, accessed on 16 December 2022 ) and following the methodology adopted by [109]. The analysis was conducted in two steps; 1. the cursors of the time window were set automatically (and then visually/manually corrected if needed) at the beginning and the end of the task to measure the total session time; 2. cursors were set at the beginning and end of each breath group (i.e., each period during which pseudowords were repeated during a single outbreath) to determine speech durations (in ms) across the task.

#### 2.2.3. Phonemic Awareness Task (Initial Phoneme Deletion Task)

In this computerized task, participants heard pseudowords consisting of three phonemes (consonant-consonant-vowel structure; for example, /spo/, /djan/) through headphones and had to orally produce the phonemic sequence obtained after deleting the first phoneme (e.g., /po/, /jan/). Pseudowords were used in order to avoid the activation of lexical knowledge. The task had to be completed as fast and as accurately as possible. Twenty-nine monosyllabic pseudowords with a CCV (consonant-consonant-vowel) structure were selected. The response time, the time taken to complete each item (processing time), and accuracy (i.e., percentage of correct responses) were measured. We calculated efficiency scores that took account of both accuracy (A) and mean response times (RT): (A/RT). Two conditions were used for this initial phoneme deletion task:

∗ *Classical Phoneme awareness task (Classical PAT)*—In this condition, the task was administered under time pressure, with the participants being required to give the answer as quickly and accurately as possible;
∗ *Delayed Phoneme awareness task (Delayed PAT)*—In this second condition, a longer interval was allowed between the end of pseudoword presentation and the response in order to facilitate access to phonemic representations of the pseudoword strings to be processed and the programming of the oral response. This interval had previously been determined for each participant individually by averaging the participant’s response time across a control repetition task consisting of 29 phonemes. In this control task, participants were asked to repeat the heard pseudowords of the PAT as accurately and as rapidly as possible. The time from the end of the audio stimulus to the end of the response was calculated and averaged for each participant. At the end of this extended time interval, a question mark appeared on the screen and the participants were able to give their answer.

The task order (Classical PAT and Delayed PAT) was counterbalanced between subjects to prevent any order effect. The list of items can be found in Appendix A.

## 3. Results

Statistical analyses were performed using the statistical open-source software packages JASP [110] and JAMOVI [111].

**Figure 1:**
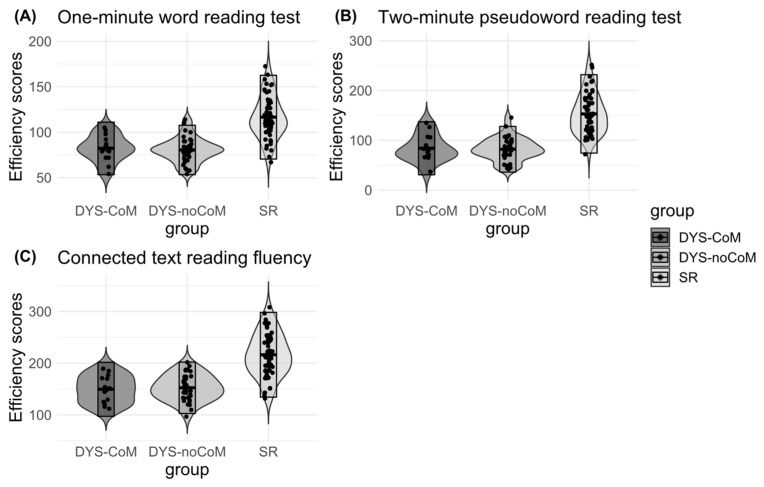
Reading performances of the three groups in three tests (**A**) One-minute word reading test; (**B**) Two-minute pseudo word reading test; (**C**) Connected text reading fluency) with mean, standard deviation, and sample points. Violin plots have the advantage of depicting the distribution of the data as well as their probability density. SR: Skilled readers, DYS-noCoM: Dyslexic readers without motor impairment), DYS-CoM: Dyslexic readers with motor impairment.

Given the percentage of individuals with both dyslexia and a motor impairment in our sample (27%), we first tested whether the two groups with dyslexia shared the same reading skills profiles. Analyses of variance (ANOVA) were conducted for each reading test (connected text reading fluency, one-minute word reading test, two-minute pseudoword reading test) and were performed with a one-way independent group (SR vs. DYS-noCoM vs. DYS-CoM) design. The dependent variable was the efficiency score in each case. The reading skills performances of the different groups are summarized in Figure 1.

The ANOVA run on the one-minute word reading performances (Figure 1A) yielded a significant effect of Group (F(2, 108) = 48.35, *p* < 0.001, η^2^ = 0.47). The contrast analyses indicated that DYS-noCoM performance did not differ from DYS-CoM (*t*(108) = 0.286, *p* = 0.678) and that the two DYS groups differed significantly from the SR group (*t*(108) = 9.089, *p* < 0.001).

The ANOVA on the two-minute pseudoword reading performances (Figure 1B) yielded a significant effect of Group (F(2, 108) = 64.710, *p* < 0.001, η^2^ = 0.55). The contrast analyses indicated that DYS-noCoM performance did not differ from DYS-CoM (*t*(108) = 0.200, *p* = 0.84), and that the two DYS groups differed significantly from the SR group (*t*(108) = 10.564, *p* < 0.001).

Finally, the ANOVA on the connected text reading fluency performances (Figure 1C) yielded a significant effect of Group (F(2, 108) = 49.876, *p* < 0.001, η^2^ = 0.48). The contrast analyses indicated that DYS-noCoM performance did not differ from DYS-CoM (*t*(108) = 0.273, *p* = 0.785), and that the two DYS groups differed significantly from the SR group (*t*(108) = 9.432, *p* < 0.001).

Overall, the results showed that the reading performances of the two groups of participants with dyslexia did not vary depending on the presence or absence of a motor coordination disorder.

### 3.1. Does Motor Impairment Associated with Dyslexia also Involve Articulatory Movements?

**Figure 2:**
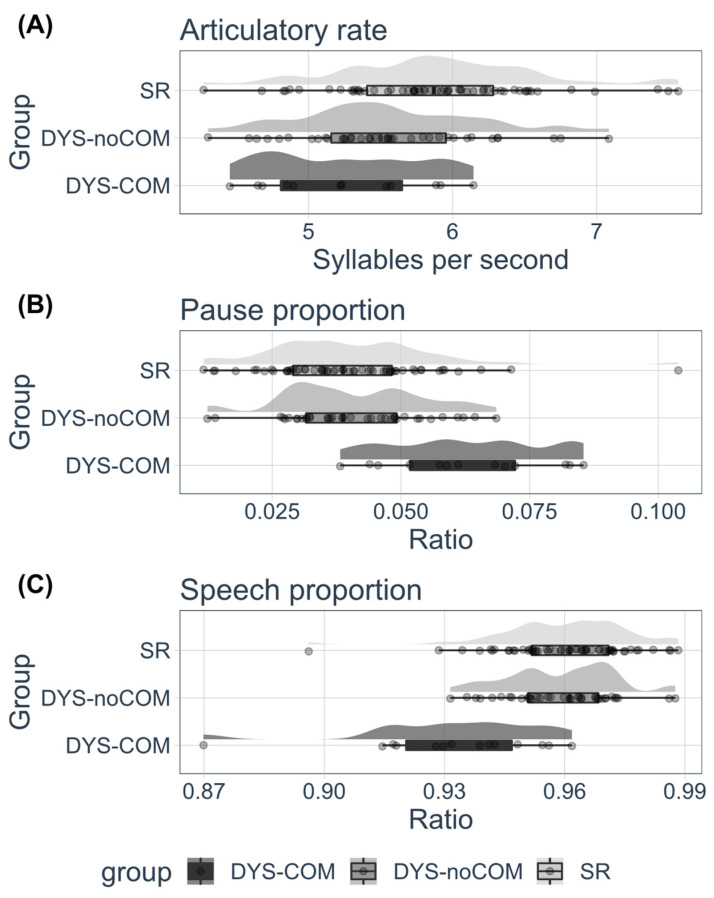
Articulatory evaluation: raincloud plots with distribution of raw data, probability density, and summary statistics (i.e., boxplot with median, interquartile ranges and confidence interval) of (**A**) “Articulatory rate”, (**B**) “Pause proportion”, (**C**) “Speech proportion” for the three groups of participants (DYS-noCoM: Dyslexic readers with no motor impairment, DYS-CoM: Dyslexic readers with motor impairment, SR: Skilled readers control group).

Descriptive raincloud plots of the articulatory task are presented in Figure 2. Raincloud plots permit the simultaneous presentation of the raw data distribution, for example probability density and summary statistics.

Since the DYS-CoM group exhibited whole-body motor impairments [49], the articulatory DDK task was administered to determine whether the motor deficit was generalized to the more complex motor skills involved in speech articulation. An ANOVA was performed on the articulatory rate, speech proportion, and pause proportion scores of the SR, DYS-COM, and DYS-noCOM groups.

The ANOVA for articulatory rate (Figure 2A) yielded a significant effect of group (F(2, 108) = 7.102, *p* = 0.001, η^2^ = 0.12). The contrast analyses indicated that DYS-noCoM performance did not differ from DYS-CoM (*t*(108) = 1.502, *p* = 0.136), and that the two DYS groups had significantly lower articulatory rates than the SR group, (*t*(108) = 3.765, *p* < 0.001). The ANOVA run on pause proportion (Figure 2B) yielded a significant effect of group (F(2, 108) = 14.128, *p* < 0.001, η^2^ = 0.21). The contrast analyses indicated that DYS-noCoM performance differed from DYS-CoM (*t*(108) = 4.716, *p* <0.001), and that the two DYS groups had significantly greater pause proportions than the SR group, (*t*(108) = 3.065, *p* < 0.001). The ANOVA on the speech proportion (Figure 2C) yielded a significant effect of group (F(2, 108) = 17.944, *p* < 0.001, η2 = 0.25). The contrast analyses indicated that DYS-noCoM performance differed from DYS-CoM (*t*(108) = 5.347, *p* <.0001), with the comorbid group exhibiting a smaller speech proportion than the DYS-noCoM participants. The two DYS groups also differed significantly from the SR group (*t*(108) = 4.423, *p* < 0.0001).

To summarize, both DYS groups had difficulties in the articulatory control of phoneme production (articulatory rate), coupled with an impaired quality of speech timing and ability to initiate speech production (speech and pause proportions). This impairment in speech timing (speech and pause proportions) was worse in the comorbid group than in theDYS-noCoM group.

### 3.2. Does Articulatory Impairment Affect the Phonemic Representations Involved in Phonemic Awareness Tasks in Dyslexia?

**Figure 3:**
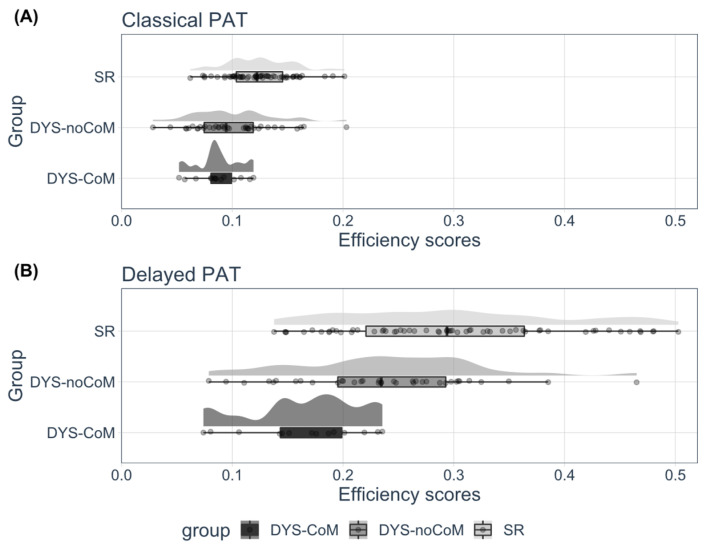
Descriptive raincloud plot of efficiency scores by group for (**A**) the Classical and (**B**) the Delayed PAT, with distribution of raw data, probability density, and summary statistics (i.e., boxplot with median, interquartile ranges, and confidence interval). SR: Skilled readers, DYS-noCoM: Dyslexic readers without motor impairment, DYS-CoM: Dyslexic readers with motor impairment.

Descriptive raincloud plots of the two phonemic awareness tasks are presented in Figure 3.

**Figure 4:**
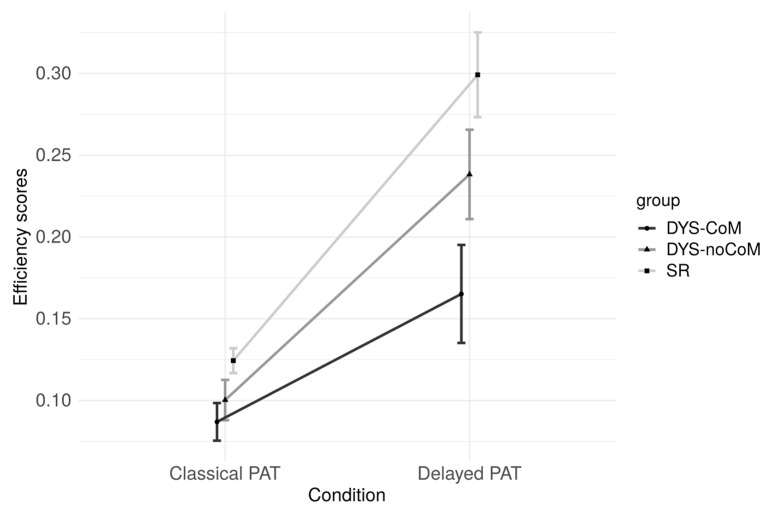
Phonemic awareness task: efficiency scores for the three groups (SR: Skilled readers, DYS-noCoM: Dyslexic readers with no motor impairment, DYS-CoM: Dyslexic readers with motor impairment), and the two conditions (Classical: Extreme time constraint, Delayed: Extended time condition).

A repeated-measures analyses of variance (ANOVA) with three groups (SR vs. DYS-noCoM vs. DYS-CoM) × 2 conditions (Classical PAT vs. Delayed PAT) mixed design was run on the efficiency scores. The results by group and by condition are presented in Figure 4. The accuracy scores in the classical and delayed PAT differed only slightly since they were already very high in the classical task in all three groups. The response times in the delayed PAT were much shorter than in the classical version. It was, therefore, response time that was responsible for the difference in efficiency scores in the two tasks.

The between-within ANOVA yielded significant effects of group (F(2, 108) = 18.125, *p* < 0.001, η^2^ = 0.11) and condition (F(1, 108) = 199.153, *p* < 0.001, η^2^ = 0.354). There was also a significant interaction between group and condition (F(2, 108) = 8.568, *p* < 0.001, η^2^ = 0.03). There was a significant effect of group in both conditions (Classical PAT; F(2, 108) = 11.676, *p* < 0.001; Delayed PAT; F(2, 108) = 14.867, *p* < 0.001). More precisely, for the classical PAT, the contrast analyses indicated that DYS-noCoM performance did not differ from DYS-CoM (*t*(108) = 0.665, *p* = 0.507), and that the two DYS groups differed significantly from the SR group (*t*(108) = 2.255, *p* < 0.025). For the delayed PAT, the contrast analyses indicated that DYS-noCoM performance differed from DYS-CoM (*t*(108) = 3.516, *p* < 0.001), and that the two DYS groups differed significantly from the SR group (*t*(108) = 7.205, *p* < 0.001). 

To summarize, the two groups with dyslexia performed worse than the control group in the two versions of the PAT, revealing the deficit in phonemic representations in dyslexia. Moreover, even though the two groups with dyslexia achieved similar performances in the classical PAT, the dyslexia-motor comorbid group performed significantly less efficiently than the non-comorbid group in the delayed PAT, suggesting that this group exhibits an additional difficulty associated with articulatory impairment.

### 3.3. What Is the Relationship between Articulatory and Phonemic Deficits in Adults with Dyslexia?

Finally, to identify some of the explanatory factors of the PAT scores, we conducted a series of ANCOVAs followed by simple main effects analyses with reading fluency scores, articulatory rate, and pause proportion (speech proportion and pause proportion were perfectly and negatively correlated and speech proportion was therefore discarded from the following analysis) as covariates, and the distinction between skilled readers and readers with dyslexia (including the two groups with dyslexia) as a qualitative factor. In the light of the results reported by Brèthes and collaborators [112], the reading fluency scores should explain the PAT scores of skilled readers only. Given the articulatory contribution to the PAT task, the scores of both groups of readers should be explained by the two articulatory indicators, with larger effects for readers with dyslexia. The results of these ANCOVAs, followed by simple main effects analyses, showed a significant interaction between group and reading fluency (F(1107) = 3.897; *p* = 0.05). More precisely, we observed that although there was a significant positive effect of reading fluency scores on PAT efficiency scores in skilled readers (t(107) = 2.08; *p* = 0.04), no such effect was found in readers with dyslexia (t(107) = −1.08; *p* = 0.28). Conversely, we found a negative effect of pause proportion on PAT efficiency scores in readers with dyslexia (t(107) = −2.001, *p* = 0.048), and no such effect for skilled readers (t(107) = –0.845, *p* = 0.400). These results were obtained even though the interaction between group and pause proportion did not reach significance (F(107) < 1). Finally, there was a main positive significant effect of articulatory rate on PAT efficiency scores (F(1107) = 5;79; *p* = 0.0018). However, the interaction between group and articulatory rate was not significant (F < 1).

Overall, the results showed that reading fluency and phonemic awareness are two dependent skills in skilled readers, but that this is not the case for readers with dyslexia. Furthermore, articulatory rate explained PAT scores in both the skilled and dyslexic group, while pause duration also explained the PAT scores of the latter group.

## 4. Discussion

The present study highlighted the potential impact of motor impairment associated with dyslexia on two oral language skills, as assessed by articulatory DDK and phoneme awareness tasks, the latter of which tests an ability that plays a crucial role in reading skills acquisition. More precisely, by using the DDK task to assess articulatory skills, we investigated whether motor deficits in adults with dyslexia could be generalized to the speech processing system. We found that both dyslexic groups had difficulties in the articulatory control of phoneme production (articulatory rate), as well as in the quality of speech timing and the ability to initiate speech production (speech and pause proportions). Moreover, speech timing (speech and pause proportions) was more severely impaired in the comorbid group than in the DYS-noCoM group.

The second aim of the study was to investigate the potential impact of articulatory impairment on phonemic awareness skills in dyslexia. With a delayed initial phoneme deletion task, we found that the comorbid group had a lower efficiency score than the non-comorbid group, while these two groups did not differ in the classical version of the task (under time pressure). Moreover, the ANCOVAs we conducted showed that the PAT performances of the participants with dyslexia were explained solely by articulatory parameters, whereas those of the control group were also explained by reading fluency scores. Together, these results confirm the deficit in phoneme representation in the two groups with dyslexia and indicate a more pronounced impairment in the comorbid group due to a generalized motor deficit.

### 4.1. Articulatory Skills in Dyslexia

A major finding of this study relates to the articulatory impairment observed in the two samples of adults with dyslexia, who were tested with the oral DDK task. The diadochokinetic articulatory rate we calculated in the present study provides us with information about the motor abilities of speech articulators, and its impairment reveals motion impairments experienced by the participants or patients. The articulation rate reflects a combination of the motor execution of speech and cognitive-linguistic processing [108,109] and is a fairly good indicator of the motor programming of speech articulators. Our findings showed that both dyslexic groups had slowed articulation rates, thus providing evidence that the cognitive/motor interface is impaired in dyslexics. Interestingly, however, other variables such as speech and pause proportions (the ratio of articulatory production time relative to total speech time and the ratio of pauses relative to total speech time, respectively) permitted a more fine-grained differentiation between the dyslexic groups, with the comorbid group exhibiting less articulatory production (and consequently, more pauses) than the group without motor comorbidity. Articulatory timing, that is, the temporal programming of articulatory movements as reflected by speech and pause proportions, appears to be considerably more impaired in the comorbid group. This suggests that the cognitive/motor interface is more severely disrupted in the comorbid group, since respiratory control and (temporal) pneumo-phonic coordination are much worse in this group.

These results are consistent with those previously reported in children and adults with dyslexia [84,85,86,87,89,113,114]. However, these earlier studies did not investigate the whole-body motor profile of the participants, making it difficult to ascertain whether these outcomes were driven by a subgroup of participants with more global motor impairment or whether this was a general characteristic of the whole dyslexic population. Our results emphasize the presence of a generalized motor disorder in dyslexic adults, with comorbidity affecting the whole body and the articulatory and respiratory control systems involved in phoneme production. By contrast, in dyslexic adults without motor comorbidity, only laryngeal and supra-laryngeal control (articulatory system) of speech sound production appears to be affected, as it also is in the comorbid group. Laryngeal control is involved when voiced phonetic features are to be produced, whereas supralaryngeal control takes place, for example, when place features (labial, dorsal, coronal) are to be produced [61]. As it can be argued that articulation features contribute to shaping phoneme representation [59,91], the results of the DDK task suggest that these phonemic representations themselves may be impacted in dyslexia rather than simply their speed of activation. Comorbid adults exhibited additional respiratory control difficulties when programming successive /pataka/ sequences. Thus, as suggested by [22], a motor impairment would affect the articulatory codes of phonemes and, consequently, the quality of the phoneme representations in memory.

These results go further those reported in adults by [87] using the NEPSY sentence repetition task, which does not permit an individualized assessment of articulatory skills, such as is possible using the DDK task. In this study, the authors distinguished groups of adults with dyslexia from those with a non-dyslexic neurodevelopmental disorder (e.g., autism spectrum disorder, specific learning disabilities, dyspraxia). However, the fact that the dyslexic group was heterogeneous and included adults with dyspraxia (9 out of 49) made the generalization of the observed articulatory deficit problematic for the interpretation of dyslexia symptoms. In the case of children with dyslexia, either the distinction between these two subgroups has not been made (e.g., [86]) or, where it has been made, articulatory skills have not been assessed [92]. Indeed, as [87] pointed out, it is necessary to go beyond diagnostic categories and address the question of the precise identification of the cognitive profiles of adults with neurodevelopmental disorders. In a similar vein, our data suggest that in addition to performing an overall evaluation of patients, it is also necessary to determine their motor profiles.

### 4.2. Phonemic Representations and Articulatory Skills in Dyslexia

The earlier studies cited in the Introduction support the hypothesis that articulatory speech skills make it possible to index the quality of phonemic representations required in different tasks involving some kind of phonemic manipulation, such as the phonemic awareness task. As such, the phonemic awareness task is thought to provide evidence highlighting a phoneme processing deficit when participants are required to consciously manipulate phonemes embedded in pseudoword sequences. This manipulation is based on speech perception skills (activated unconsciously) such as identification and discrimination abilities which permit the conscious manipulation of phonemes [115]. From a developmental point of view, [116] followed children with neurotypical development between the ages of 4 and 5 and reported that phoneme articulation accuracy predicted speech perception and phoneme awareness skills after age, vocabulary, and letter-word knowledge were controlled for. According to the authors, these findings are consistent with a model in which children’s articulation accuracy affects pre-existing differences in phonological representations and, consequently, affects how children perceive, discriminate, and manipulate speech sounds (see [91] for similar results).

The poorer performance of both dyslexia groups compared to the control group in both modalities of the PAT replicates the numerous findings of persistent deficits in phonemic awareness in dyslexic adults [14,15,117]. Although the performance of the two groups did not differ in the classical PAT, the performance of the comorbid group was lower than that of the non-comorbid group in the delayed PAT, suggesting that the additional time provided to permit full access to phonemic representations brought about a smaller efficiency gain in this latter group. Furthermore, both dyslexic groups exhibited a shared deficit in articulatory control during phoneme production (articulatory rate), and the comorbid group also exhibited a general respiratory control deficit (speech proportion and pause duration). These results suggest that access to imprecise or degraded phonemic representations is partly related to dimensions of the motor control of speech. Respiratory control impairment seems to constitute an additional difficulty, as suggested by the greater impact on speech and pause proportions in the comorbid group. This possibility needs to be tested more directly in further studies. Indeed, we cannot totally rule out the hypothesis that articulatory disorders might also affect the production of the response itself, given that the results of the conducted ANCOVAs showed that the delayed PAT scores were explained by the articulatory performance of both groups, that is, with and without dyslexia. However, the results of the ANCOVAs also showed that the delayed PAT scores of the skilled readers were additionally explained by their reading fluency scores, which modulated the effect of articulatory factors compared to adults with dyslexia. In these latter participants, the PAT scores were explained solely by phoneme production factors and were independent of their reading fluency scores. These latter findings are consistent with studies reporting that the reading skills of adults with dyslexia are not underpinned by their phonological (and therefore phonemic) skills, unlike in the case of skilled adult readers [14,112,117,118]. The hypothesis according to which impaired articulatory speech skills influence phonemic representations in dyslexia is consistent with the results of studies that have provided some articulatory training for dyslexia remediation and reported a reduction in the phonological disorder coupled with an improvement in reading and spelling performance [119,120,121].

### 4.3. Limitations

The above-chance incidence of comorbid literacy/dyslexic and motor difficulties reported in previous experiments and in the present study supports the claim that these disorders are, to some extent, related [5,10]. However, the nature of the association between literacy and motor disorders remains poorly understood. We have demonstrated a specific profile of articulatory deficits that affects phoneme representations in comorbid individuals with dyslexia. However, the direct relationship between articulatory skills impairment and reading skills needs to be addressed in future research, as our results provide no evidence of a direct link. We showed that motor, and more precisely, articulatory impairment, may only be a distal factor of reading deficits in dyslexia, as recent finding with children [92] and a proposal by [122] may suggest. Finally, a DCD control group should be included in future research in order to identify the impact of dyslexia on the motor deficit profiles more clearly.

## Data Availability

The data presented in this study are available on request from the corresponding author at pascale.cole@univ-amu.fr.

## Appendix A

List of the 29 pseudowords used in the Classical and delayed PAT.

***Stimuli***
djan
dron
flin
fra
gle
gna
grou
klo
kron
ksan
plou
pra
pso
pson
sfe
skin
sla
spon
snou
spu
sri
stan
sti
tcheu
tra
tse
vlon
vri
vron
